# Specific risk factors contributing to early and late recurrences of intrahepatic cholangiocarcinoma after curative resection

**DOI:** 10.1186/s12957-018-1540-1

**Published:** 2019-01-03

**Authors:** Changzheng Wang, Shujie Pang, Hui Si-Ma, Ning Yang, Haibin Zhang, Yong Fu, Guangshun Yang

**Affiliations:** Department of Hepatic Surgery, Eastern Hepatobiliary Surgery Hospital, Second Military Medical University, No. 225,Changhai Road, Shanghai, 200433 China

**Keywords:** Cholangiocarcinoma, Hepatectomy, Prognosis, Recurrence, Risk factor

## Abstract

**Background:**

Most intrahepatic cholangiocarcinoma (ICC) patients experienced tumor recurrences even after curative resection, but the optimal cut-off time point and the specific risk factors for early and late recurrences of ICC have not been clearly defined. The objective of the current study was to define specific risk factors for early and late recurrences of ICC after radical hepatectomy.

**Methods:**

Included in this study were 259 ICC patients who underwent curative surgery at our hospital between January 2005 and December 2009. Recurrences in these patients were followed-up prospectively. Piecewise regression model and the minimum *P* value approach were used to estimate the optimal cut-off time point for early and late recurrences. Then, Cox’s proportional hazards regression model was used to identify specific independent risk factors for early and late recurrences.

**Results:**

Early and late recurrences occurred in 130 and 74 patients, respectively, and the 12th month was confirmed as the optimal cut-off time point for early and late recurrences. Cox’s proportional hazards regression model showed that microvascular invasion (HR = 2.084, 95% CI 1.115–3.897, *P* = 0.021), multiple tumors (HR = 2.071, 95% CI 1.185–3.616, *P* = 0.010), abnormal elevation of serum CA19-9 (HR = 1.619, 95% CI 1.076–2.437, *P* = 0.021), and the negative hepatitis B status (HR = 1.650, 95% CI 1.123–2.427, *P* = 0.011) were independent risk factors for early recurrence, and HBV-DNA level > 10^6^ IU/mL (HR = 1.785, 95% CI 1.015–3.141, *P* = 0.044) and a hepatolithiasis history (HR = 2.538, 95% CI 1.165–5.533, *P* = 0.010) contributed to late recurrence independently.

**Conclusion:**

Specific risk factors and mechanisms may relate to early and late recurrences of ICC after curative resection.

## Background

Intrahepatic cholangiocarcinoma (ICC) ranks the second most common malignancy of the liver, accounting for 10–15% of all primary liver cancers with an increasing trend of new incidences annually [1]. Hepatectomy has been recognized as the first-line treatment for ICC. However, the prognosis of ICC patients remains dismal due to the aggressive biological behavior of ICC even after curative resection [2, 3]. Previous studies reported that the 5-year recurrence rate was 54.3–77.4% for ICC patients after curative hepatectomy, with a median survival time of 10.5–18.7 months [4–9]. In addition, intrahepatic recurrence is the most common form of recurrence, followed by intrahepatic recurrence combined with extrahepatic recurrence and exclusive extrahepatic recurrence [10–12].

So far, various factors have been found to be associated with postoperative ICC recurrence, including microvascular invasion, preoperative lymph node metastasis, multifocal involvement, and elevation of serum carbohydrate antigen 19-9 (CA19-9) and γ-glutamyl transpeptidase (γ-GT) levels [8, 13]. Some recent studies [14–17] suggested that specific risk factors and mechanisms were involved in early and late recurrences for hepatocellular carcinoma (HCC) patients after liver resection, reporting that microsatellite, microvascular invasion (MVI), and abnormal elevation of alpha-fetoprotein (AFP) were associated with early recurrence, while cirrhosis and hepatitis activity contributed to late recurrence in HCC. It was therefore postulated that early recurrence might derive from intrahepatic metastasis, while late recurrence was most likely due to multicentric occurrence. Although, risk factors contributing to early or late recurrences of HCC have been studied extensively, specific factors contributing to either early or late recurrence in ICC remain unclear. Data concerning risk factors related to early or late recurrences of ICC may have significant implications for postoperative surveillance after curative resection, knowing that ICC patients with specific risk factors may need different followed-up protocols. The current study aimed to identify specific risk factors for early and late recurrences in ICC patients after radical hepatectomy, and suggest individualized followed-up protocols for ICC patients with specific risk factors.

## Material and methods

### Patients

Enrolled in the current study were 259 ICC patients who underwent curative hepatectomy at the Eastern Hepatobiliary Surgery Hospital (Shanghai, China) between January 2005 and December 2009. Preoperative diagnoses were based on contrast-enhanced CT or contrast-enhanced MRI findings and serological tumor makers AFP, CA19-9, and carcinoembryonic antigen (CEA). Postoperative diagnoses were confirmed by pathology. It is worth mentioning that quite a few patients with preoperative elevation of serum AFP level were misdiagnosed with HCC, which was later corrected by postoperative pathology. The indications for ICC patients to receive curative liver resection were (1) child’s classification of liver function of A or B, (2) tumors involving no more than three liver segments, (3) the absence of portal vein main trunk involvement and extrahepatic metastasis, and (4) the preoperative WHO performance status of 0–1. Curative resection was defined as complete excision of the tumor with a negative microscopic margin and no residual lesion detected by CT or MR imaging a month after surgery. The inclusion criteria for patients in this study were (1) primary liver cancer with no previous history of therapy before surgery, (2) ICC confirmed by postoperative histology, (3) meeting the indications and criteria of curative resection mentioned above, and (4) ICC patients with detailed information with respect to clinicopathology and recurrence. The exclusion criteria were (1) liver lesions confirmed to be combined HCC plus ICC, (2) patients who died during the follow-up period of other causes except tumor recurrence, and (3) patients with incomplete data or beyond the criteria of curative resection.

### Analysis of variables

All potential risk factors for early or late recurrences were divided into three groups: host-related, serum makers, and tumor-related. With regard to host-related factors, we assessed gender, age (under or over 60 years), the liver state (cirrhotic or normal), liver function (Child A or B), and history of hepatitis B virus (HBV) infection and hepatolithiasis. HBV infection was defined as HBsAg (+) or hepatitis B virus deoxyribonucleic acid (HBV-DNA) > 10^3^ IU/mL. Of the serum makers, we analyzed total bilirubin (TBIL) (≤ 17.1 or > 17.1 μmol/l), alanine aminotransferase (ALT) (≤ 50 or > 50 U/l), alkaline phosphatase (ALP) (≤ 119 or >  U/L), aspartate aminotransferase (AST) (≤ 37 or > 37 U/L), γ-GT(≤ 64 or > 64 U/L), CA19-9(≤ 37 or > 37 U/L), AFP (≤ 20 or > 20 μg/L), CEA (≤ 10 or > 10 μg/L), and the status and level of preoperative HBV-DNA (≤ 10^6^ or > 10^6^ IU/mL). Of the tumor-related factors, we investigated the number of tumors (single or multiple), diameter (more or less than 5 cm), tumor differentiation grade (high, moderate, or poor), macroscopic and microscopic vascular invasion (yes or no), integrity of the tumor capsule (yes or no), lymphatic metastasis (yes or no), TNM classification (I, II, or III), and satellite lesions (yes or no).

### Follow-up observations

After hospital discharge, the patients were followed-up regularly in the outpatient clinic using a standard protocol every 3 months during the first 2 years, and twice a year afterwards. Follow-up observations included serum level of AFP, CEA, and CA19-9, and abdominal ultrasonography. If there was potential recurrence found, further CT or MRI examination was employed to confirm or exclude it. All patients were followed-up consecutively until October 2014.

This study complied with the ethical guidelines of the Declaration of Helsinki, and analyses were carried out with institutional medical ethical consent in an anonymized database.

### Statistical analysis

Piecewise regression model and minimum *P* value approach were utilized to identify the optimal cut-off time point for early and late recurrences. Continuous variables were compared with the Mann-Whitney *U* test, while Fisher’s exact test was used to compare categorical variables. For continuous variables, clinically applicable cut-offs were chosen for easy interpretation. The cumulative survival time was calculated by using the Kaplan–Meier method and compared by log-rank test. Cox’s proportional hazards regression model was employed to conduct univariate and multivariate analyses of risk factors for early and late recurrences of ICC patients. Statistical significance was defined as *P* < 0.05. All statistical analyses in this study were performed with software R version 3.0.

## Results

### Evaluation of the optimal cut-off time point for early and late recurrences

**Fig. 1:**
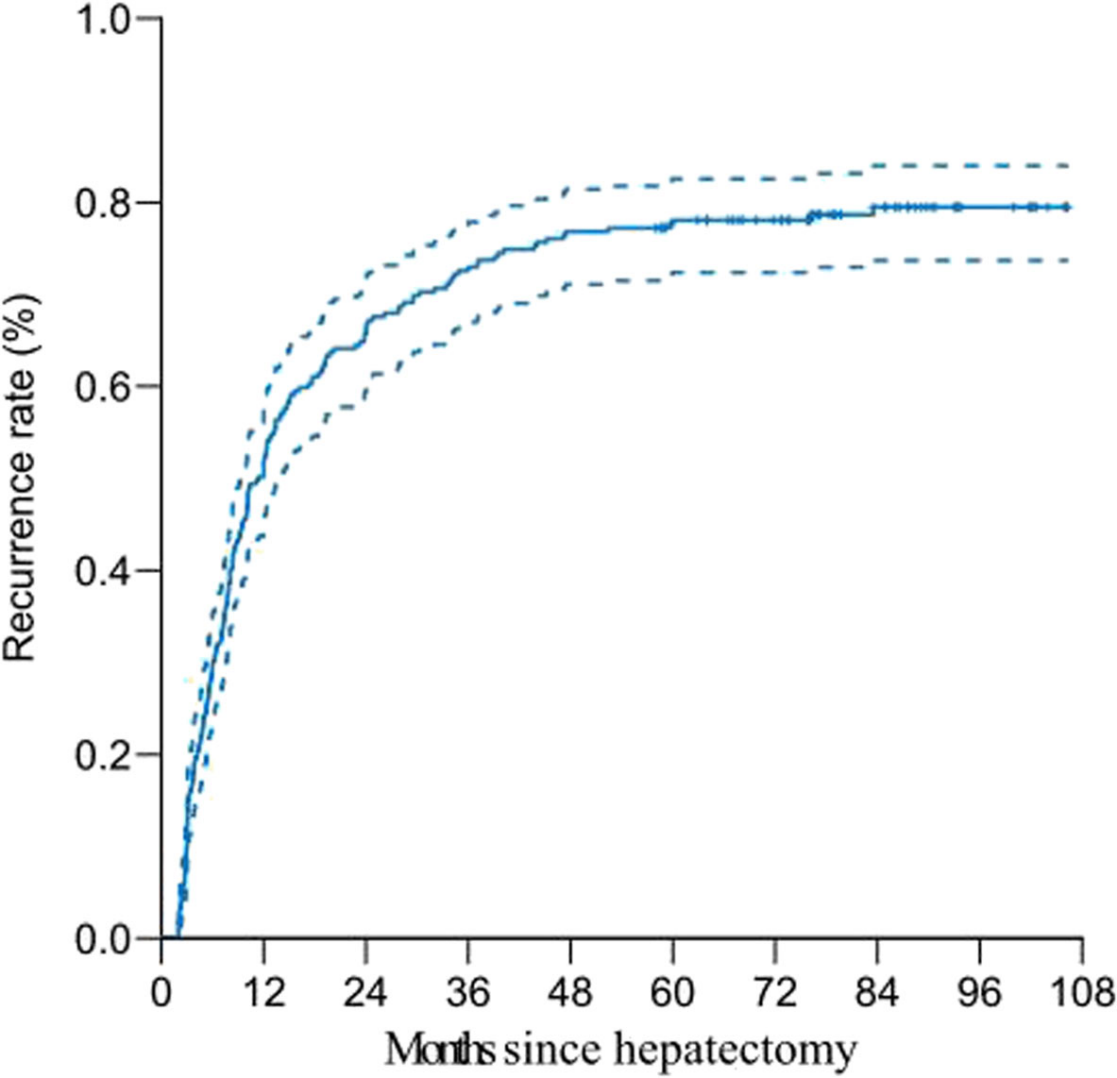
Cumulative recurrence-free survival curve of 259 ICC patients after curative hepatectomy

**Fig. 2:**
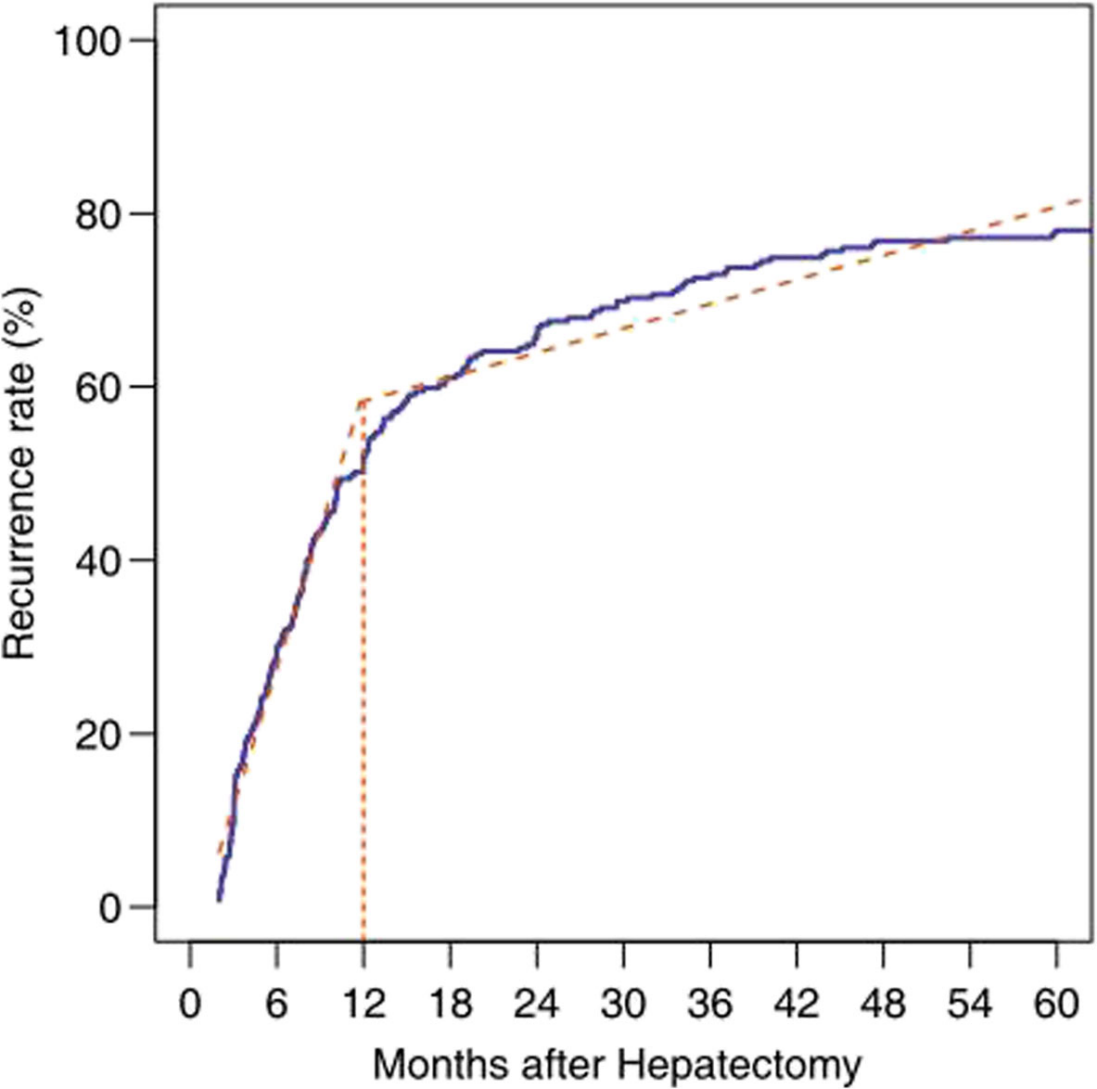
The optimal cut-off time point estimated by piecewise regression model

**Fig. 3:**
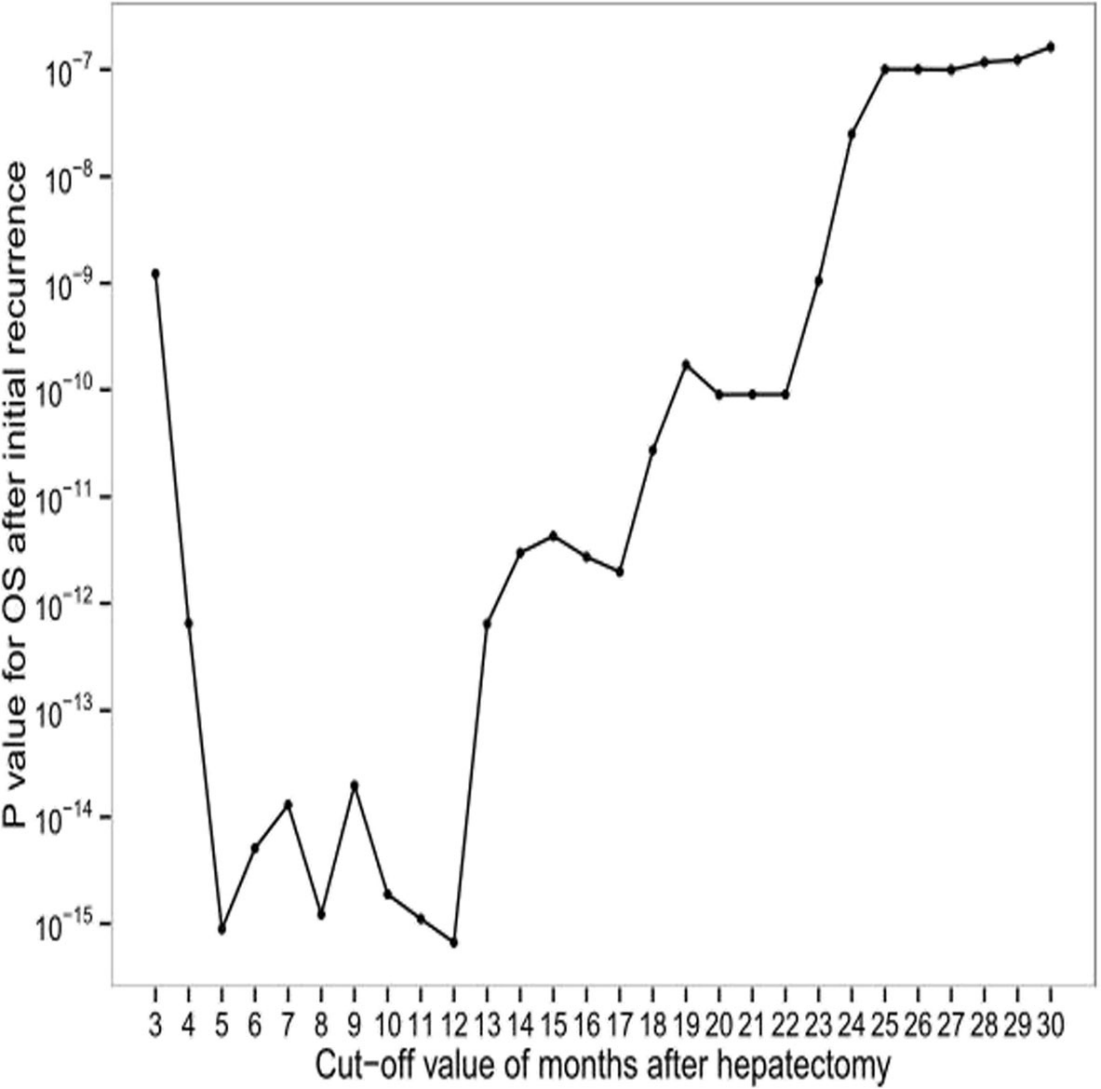
The optimal cut-off time point verified in the minimum *P* value approach

A total of 259 ICC patients were enrolled in this study, of whom 204 patients experienced tumor recurrences, with a median recurrence interval of 11.9 months. The clinicopathological characteristics of all patients are shown in Table 1, and the tumor recurrence rate for all patients is shown in Fig. 1. The 1-, 2-, and 3-year recurrence-free survival rate was 50%, 34%, and 27%, respectively. Then, we evaluated the optimal cut-off time point for differentiating early and late recurrences preliminarily by piecewise regression model. The results in Fig. 2 demonstrated that the optimal cut-off time point was approximate to the 12th month. In addition, the minimum *P* value approach was utilized to verify this cut-off point with the minimum *P* value of 10^−5^ on the 12th month (Fig. 3). In two different ways, we obtained a consistent cut-off point, based on which we defined the 12th month as the optimal cut-off time point for early and late recurrences for ICC patients who underwent curative surgery.

**Table 1:** Clinicopathological characteristics of all patients

Variable	Number	Percentage (%)
Gender		
Male vs. female	174/85	67.2/32.8
Age (year)	55	
HBV infection		
Positive vs. negative	137/122	52.9/47.1
HBV-DNA load (IU/mL)		
≥ 10^3^ vs. < 10^3^	59/200	22.8/77.2
ALT (U/L)		
≤ 50 vs. > 50	220/39	84.9/15.1
AST (U/L)		
≤ 40 vs. > 40	203/56	78.4/21.6
γ-GT (U/L)		
≤ 50 vs. > 50	126/133	48.0/52.0
TBIL (μmol/L)		
≤ 17 .1 vs. > 17.1	167/92	64.5/35.5
ALP (U/L)		
≤ 150 vs. > 150	178/81	68.7/31.3
Cirrhosis		
Yes vs. no	82/217	31.7/68.3
Child-Pugh class		
A vs. B	247/12	95.3/4.7
AFP (ng/L)		
≤ 20 vs. > 20	210/49	81.1/18.9
CEA (μg/L)		
≤ 10 vs. > 10	239/20	92.3/7.7
CA19-9 (U/L)		
≤ 37 vs. > 37	124/135	47.9/52.1
Tumor size (cm)		
≤ 5 vs. > 5	121/138	46.7/53.3
Tumor number		
Single vs. multiple	219/40	84.6/15.4
Satellite		
No vs. yes	159/100	61.4/38.6
Capsule integrity		
Yes vs. no	14/245	5.4/94.6
Macrovascular invasion		
Yes vs. no	18/241	7.0/93
Microvascular invasion		
Yes vs. no	12/247	4.7/95.3
E-S grade		
I vs. II vs. III	4/222/33	1.6/85.7/12.7
LNM		
Yes vs. no	40/219	15.5/84.5
TNM stage		
I or II vs. III	226/33	87.3/12.7

*Abbreviations*: *HBV-DNA* hepatitis B virus deoxyribonucleic acid, *ALT* alanine aminotransferase, *AST* aspartate aminotransferase, *γ-GT* γ-glutamyl transpeptidase, *ALP* alkaline phosphatase, *TBIL* total bilirubin, *AFP* alpha-fetoprotein, *CEA* carcinoembryonic antigen, *CA19-9* carbohydrate antigen 19-9, *E-S* Edmondson-Steiner, *LNM* lymph node metastasis

### Risk factors for early recurrence

Early recurrence was observed in 130 patients. Cox proportional hazards model is to link the survival time of an individual to the covariates, which makes it possible to find out the most important covariate impact on the survival time of a patient. Thus, Cox’s proportional hazards regression model was used to identify independent risk factors for early recurrence. Univariate analysis showed that tumor size, tumor number, satellite nodules, macro- and microvascular invasion, an advanced TNM stage, HBV infection, and elevation of serum ALP, γ-GT, and HBV-DNA levels were related to early recurrence (Table 2). These variates were then subjected to the multivariate analysis, and the result showed that microvascular invasion, multiple tumors, elevation of serum CA19-9, and the negative HBV status were independent risk factors (Table 3) for early recurrence of ICC patients after curative hepatectomy.

**Table 2:** Univariate analysis of factors related to early recurrence

Variable	HR	95% CI	*P* value
Gender			
Male vs. female	0.914	0.628–1.330	0.641
Age (year)	0.998	0.983–1.014	0.827
ALT (U/L)	1.148	0.726–1.816	0.799
AST (U/L)	1.138	0.760–1.704	0.527
ALP (U/L)	1.853	1.306–2.629	*0.001*
γ-GT (U/L)	1.613	1.135–2.291	*0.008*
TBIL (μmol/L)	1.229	0.813–1.858	0.325
HBV infection			
Positive vs. negative	0.513	0.361–0.728	*< 0.001*
HBV-DNA load (IU/mL)			
≥ 10^3^ vs. < 10^3^	0.591	0.373–0.934	*0.023*
HBV-DNA load (IU/mL)			
≥ 10^6^ vs. < 10^6^	0.439	0.205–0.941	*0.029*
Cirrhosis			
Yes vs. no	0.733	0.496–1.084	0.118
Child-Pugh class			
B vs. A	0.942	0.415–2.138	0.887
Hepatolithiasis history			
Yes vs. no	2.149	1.377–3.355	*0.001*
AFP (ng/mL)			
> 20 vs. ≤ 20	0.844	0.534–1.336	0.468
CEA (μg/L)			
> 10 vs. ≤ 10	2.498	1.497–4.169	*< 0.001*
CA19-9 (U/L)			
> vs. ≤ 37	2.409	1.670–3.475	*< 0.001*
Tumor size (cm)			
> 5 vs. ≤ 5	1.982	1.385–2.837	*0.001*
Tumor number			
Multiple vs. single	3.110	2.080–4.650	*< 0.001*
Satellite			
Yes vs. no	2.491	1.737–3.573	*< 0.001*
Macrovascular invasion			
Yes vs. no	2.215	1.290–3.801	*0.003*
Microvascular invasion			
Yes vs. no	3.035	1.629–5.656	*< 0.001*
Capsule integrity			
Yes vs. no	0.801	0.353–1.818	0.591
E-S grade			
III vs. I or II	1.468	0.995–2.167	0.053
LNM			
Yes vs. no	1.591	0.943–2.686	0.082
TNM stage			
III vs. II or I	1.952	1.243–3.066	*0.004*

*Abbreviations*: *ALT* alanine aminotransferase, *AST* aspartate aminotransferase, *ALP* alkaline phosphatase, *γ-GT* γ-glutamyl transpeptidase, *TBIL* total bilirubin, *HBV-DNA* hepatitis B virus deoxyribonucleic acid, *AFP* alpha-fetoprotein, *CEA* carcinoembryonic antigen, *CA19-9* carbohydrate antigen 19-9, *E-S* Edmondson-Steiner, *LNM* lymph node metastasis. There were statistically significant differences for data in Italics (p < 0.05)

**Table 3:** Multivariate analysis of factors related to early recurrence

Variable	HR	95% CI	*P* value
Microvascular invasion			
Yes vs. no	2.084	1.115–3.897	*0.021*
Tumor number			
Multiple vs. single	2.071	1.185–3.616	*0.010*
CA19-9 (U/L)			
> 37 vs. ≤ 37	1.619	1.076–2.437	*0.021*
HBV infection			
Positive vs. negative	1.650	1.123–2.427	*0.011*

*Abbreviations*: *CA19-9* carbohydrate antigen 19-9, *HBV* hepatitis B virus. There were statistically significant differences for data in Italics (p < 0.05)

### Risk factors for late recurrence

Recurrence after 12 months occurred in 74 patients. Both univariate and multivariate analyses showed that serum HBV-DNA load > 10^6^ IU/mL and a hepatolithiasis history contributed to late recurrence independently (Tables 4 and 5).

**Table 4:** Univariate analysis of factors related to late recurrence

Variate	HR	95% CI	*P* value
Gender			
Male vs. female	1.206	0.745–1.954	0.445
Age (year)	1.000	0.981–1.020	0.986
ALT (U/L)	1.144	0.602–2.172	0.682
AST (U/L)	1.365	0.794–2.347	0.258
ALP (U/L)	1.336	0.785–2.273	0.205
γ-GT (U/L)	1.219	0.771–1.929	0.397
TBIL (μmol/L)	1.485	0.862–2.556	0.154
HBV infection			
Positive vs. negative	0.587	0.298–1.157	0.124
HBV-DNA load (IU/mL)			
≥ 10^3^ vs. < 10^3^	1.145	0.703–1.865	0.587
HBV-DNA load (IU/mL)			
≥ 10^6^ vs. < 10^6^	1.784	1.023–3.111	*0.038*
Cirrhosis			
Yes vs. no	0.910	0.567–1.461	0.698
Child-Pugh class			
B vs. A	1.283	0.468–3.516	0.625
Hepatolithiasis history			
Yes vs. no	2.661	1.265–5.599	*0.007*
AFP (ng/mL)			
> vs. ≤ 20	0.781	0.429–1.422	0.416
CEA (μg/L)			
> 10 vs. ≤ 10	3.555	1.107–11.412	*0.022*
CA19–9 (U/L)			
> 37 vs. ≤ 37	1.331	0.836–2.117	0.228
Tumor size (cm)			
> vs. ≤ 5	1.383	0.875–2.183	0.163
Tumor number			
Multiple vs. single	1.661	0.719–3.836	0.235
Satellite			
Yes vs. no	1.308	0.689–2.484	0.411
Macrovascular invasion			
Yes vs. no	1.176	0.288–4.800	0.819
Microvascular invasion			
Yes vs. no	1.544	0.214–11.143	0.663
Capsule integrity			
Yes vs. no	0.881	0.322–2.415	0.806
E-S grade			
III vs. I or II	0.895	0.174–4.616	0.311
LNM			
Yes vs. no	1.355	0.546–3.362	0.509
TNM stage			
III vs. I or II	1.483	0.680–3.232	0.317

*Abbreviations*: *ALT* alanine aminotransferase, *AST* aspartate aminotransferase, *ALP* alkaline phosphatase, *γ-GT* γ-glutamyl transpeptidase, *TBIL* total bilirubin, *HBV-DNA* hepatitis B virus deoxyribonucleic acid, *AFP* alpha-fetoprotein, *CEA* carcinoembryonic antigen, *CA19-9* carbohydrate antigen 19-9, *E-S* Edmondson-Steiner, *LNM* lymph node metastasis. There were statistically significant differences for data in Italics (p < 0.05)

**Table 5:** Multivariate analysis of factors related to early recurrence

Variable	HR	95% CI	*P* value
Hepatolithiasis history			
Yes vs. no	2.538	1.165–5.533	*0.010*
HBV-DNA load (IU/mL)			
≥ vs. < 10^6^	1.785	1.015–3.141	*0.044*

*Abbreviations*: *HBV-DNA* hepatitis B virus deoxyribonucleic acid. There were statistically significant differences for data in Italics (p < 0.05)

### Difference in overall survival (OS) between early- and late-recurrence ICC patients

**Fig. 4:**
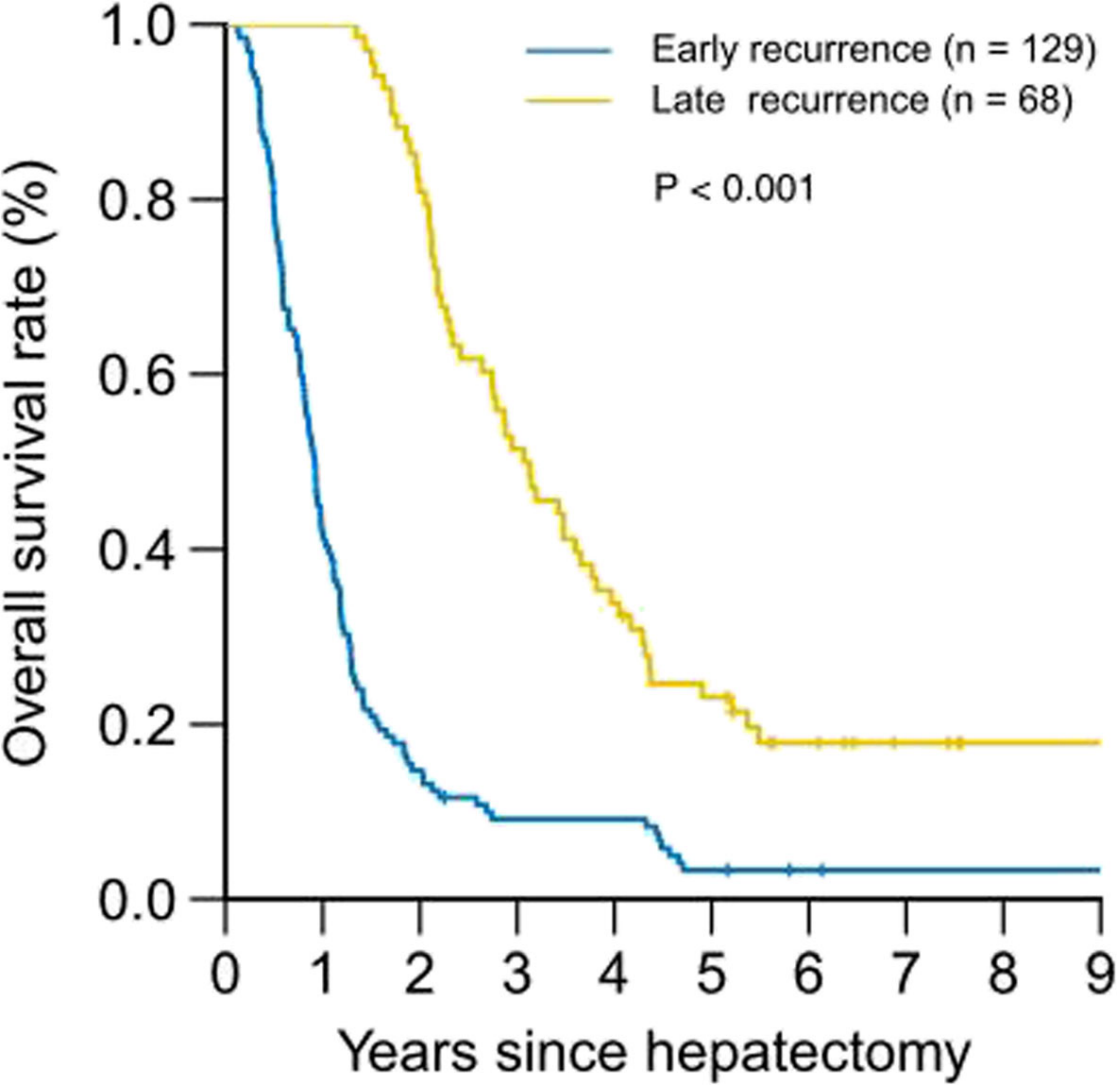
OS of ICC patients with early or late recurrence (*p* < 0.001)

OS differences between early- and late-recurrence ICC patients are shown in Fig. 4. The 1-, 3-, and 5-year OS rate for early- and late-recurrence patients was 45% vs. 100%, 8% vs. 50%, and 3% vs. 21%, respectively (*p* < 0.001). Seven patients were lost to follow-up after tumor recurrence, one in early-recurrence group, and six in late-recurrence group.

## Discussion

With the advancement in the technique and theory of hepatic surgery, hepatectomy has been regarded as an effective and safe procedure for primary liver cancer patients [18, 19]. But as there is a high recurrence rate after surgery, the prognosis for primary liver cancer patients remains dismal, especially for ICC patients [1, 2, 18, 19]. Previous studies [14, 16] identified time interval from hepatectomy to recurrence as an independent risk factor of OS for HCC patients. Specific risk factors and mechanisms may be involved in early and late recurrences of primary liver cancer after curative therapies. Some studies on HCC [14–17] have suggested some specific factors contributing to early and late intrahepatic recurrences. They found that early tumor recurrence was mostly associated with tumor-related factors, including microvascular invasion, multiple tumors, and elevation of serum AFP level, while late tumor recurrence was most associated with host-related factors, including liver fibrosis and hepatitis activity. They therefore postulated that early tumor recurrence was combined to intrahepatic metastasis and late tumor recurrence might originate from the remnant liver after hepatectomy. The present study intended to identify the risk factors and mechanisms underlying early and late recurrences in ICC patients after curative liver resection.

Some previous studies [15–17] set the 24th month as the optimal cut-off time point for early and late recurrences of HCC patients after curative hepatectomy, which is consistent with the opinion of Zhang et al. [20], who also set the 24th month as the optimal cut-off time point for early and late recurrence of ICC patients after curative resection. Given the aggressive biological characteristics compared with HCC, we suggest the optimal cut-off time point for early and late recurrences should be moved up to an early date. The piecewise regression model and minimum *P* value approach in our study seemed to suggest that the 12th month was the optimal dividing point for early and late postoperative recurrences of ICC patients. In addition, compared with HCC, ICC tends to develop lymphatic metastasis and intrahepatic metastasis in earlier stages [1, 2], and ICC patients usually had a more dismal prognosis than HCC patients. Thus, we divided these postoperative recurrence ICC patients into early- and late-recurrence groups by 12 months.

Our study revealed that multiple tumors, lymph node metastasis, elevation of serum CA19-9 level, and a negative hepatitis B status were independent risk factors associated with early recurrence. Previous studies in HCC patients reported tumor-related factors including microvascular invasion, multiple tumors, lymph node metastasis, and elevation of preoperative serum AFP level were contributing factors of early tumor recurrence, which is consistent with the finding of the present study. These aggressive tumor-related factors are likely to lead to residual tumors or intrahepatic micrometastasis, which could not be detected by contemporary imaging techniques after surgery, and therefore may result in intrahepatic metastasis in future. Elevation of preoperative serum CA19-9 level was also identified as an independent risk factor for early recurrence. Recent studies [21, 22] showed that elevation of serum CA19-9 was related to a high tumor burden and predicted a negative prognosis for ICC patients after surgery. It is interesting to find in the current study that the negative status of hepatitis B was an independent factor contributing to early tumor recurrence; in other words, hepatitis B activity was a favorable prognostic factor for ICC patients, which is contradictory to the finding of some recent studies reporting that HBV infection increased the risk of ICC incidence [23–25]. However, we found that HBV-related ICC patients had a better prognosis compared with ICC patients without HBV infection. Some studies [25] suggested that HBV-associated ICC and HCC may share a common carcinogenetic process. Maybe, HBV-associated ICC is a special category of ICC. To sum up, our results demonstrated that tumor recurrence at an early phase was mainly due to residual microlesions or intrahepatic micrometastasis. A more regular follow-up protocol is recommended for ICC patients with these risk factors after liver resection.

Unlike factors affecting early tumor recurrence, factors related to late-phase recurrence were mainly host-related factors, including a history of hepatolithiasis and serum HBV-DNA load > 10^6^ IU/mL. While HBV infection acted as a favorable factor for tumor early phase recurrence, HBV-DNA level > 10^6^ IU/mL was associated with tumor late recurrence. However, HBV-DNA level to a large extent reflects the grade of hepatitis activity. This result was consistent with previous studies in HCC which showed that the grade of hepatitis activity was closely correlated with late-phase recurrence [15]. The present study showed that a hepatolithiasis history was another risk factor affecting late tumor recurrence. Hepatolithiasis is the main risk factor contributing to the incidence of ICC. Long-term chronic infection of the biliary tract secondary to hepatolithiasis would lead to chronic inflammation of biliary epithelial cells. In such a microenvironment, ICC was easy to revive [26–28]. These two etiology-related factors may reflect increased carcinogenicity of the background liver status after curative hepatectomy. Our findings support the hypothesis that late recurrence was mainly attributed to a second primary lesion from the remnant liver.

The current study also revealed that the interval from surgery to recurrence was a risk factor affecting the prognosis of ICC patients after curative hepatectomy. Compared with early-recurrence patients, patients in the late-recurrence group had obviously better OS.

The current study has several limitations. First, the retrospective nature of this study had its own disadvantages. There may have been selection bias in this study. Thus, prospective cohort studies and prediction models are needed to assess the recurrence phase of ICC. Second, as this was a single-center cohort study with a relatively small sample size, the results may not be generalizable. Thus, further multicenter and larger-sample studies are still necessary.

In conclusion, the current study may shed new light on the pathogenesis of early- and late-phase recurrence of ICC in patients after curative surgery. It is remarkable that early tumor recurrence is chiefly ascribed to residual microlesions or intrahepatic micrometastasis, which is closely associated with tumor-related factors, while late recurrence is mainly attributed to a second primary lesion from the remnant liver, which is closely correlated with the background of the remnant liver. We recommend that ICC patients who present multiple tumors, lymph node metastasis, elevation of serum CA19-9, and a negative hepatitis B status should be followed-up more closely and regularly after curative resection. Whether these ICC patients should undergo other adjuvant therapies after surgery remains unclear, and our future study will focus on this issue.

## Abbreviations

AFP: Alpha-fetoprotein
ALP: Alkaline phosphatase
ALT: Alanine aminotransferase
AST: Aspartate aminotransferase
CA19-9: Carbohydrate antigen 19-9
CEA: Carcinoembryonic antigen
E-S: Edmondson-Steiner
HBV: Hepatitis B virus
HBV-DNA: Hepatitis B virus deoxyribonucleic acid
HCC: Hepatocellular carcinoma
ICC: Intrahepatic cholangiocarcinoma
LNM: Lymph node metastasis
MVI: Microvascular invasion
OS: Overall survival
TBIL: Total bilirubin
γ-GT: γ-Glutamyl transpeptidase
